# Memoirs of the Medical Society of Observation of Paris

**Published:** 1845-04

**Authors:** 


### Art. V.

*Mémoires de la Société Médicale d'Observation de Paris.* Tome II.—*Paris*, 1844.

*Memoirs of the Medical Society of Observation of Paris.* Vol. II.—*Paris*, 1844. 8vo, pp. 628.

After the lapse of six years this Society has once more given indication of its existence by the volume before us. It contains four essays, the first of which, by M. Louis, on the Yellow Fever of Gibraltar, we noticed on its appearance in an English dress from the pen of Dr. Shattuck; and shall therefore not again advert to it. The second contains an account of a series of extremely well-conducted observations on the pulse in infancy and childhood, by M. Valleix. All investigations concerning the pulse need a larger amount of patient perseverance than is possessed by most, as well as very considerable acumen in avoiding sources of error which might otherwise entirely vitiate any result at which the observer might arrive. Hence, till comparatively recently, we have had little beyond mere assertion, or at best very inconclusive experiments, as the basis of our knowledge with reference to the variations of the pulse in health and disease. All the difficulties that involve this subject in the adult become doubled when the inquiry refers to infancy and childhood, and our knowledge, small as it was, with reference to the pulse in the grown subject, was incomparably less with regard to it in the child. The interesting researches of Dr. Knox and of Dr. Guy were carried on in the grown subject, those of Mr. Gorham, published some years since in the 'Medical Gazette,' and still more recently those of M. Trousseau, had reference to the young subject, but were not sufficiently numerous to be decisive. The observations of M. Billard on the pulse in infancy, were not made with due precautions, hence his results are very contradictory and inconclusive. M. Valleix's work on children's diseases, published between six and seven years ago, contains the particulars of a series of observations on the pulse in the new-born infant, which are republished here in a more extended form, with the addition of some others hitherto unpublished. The close approximation of many of his conclusions to those at which Mr. Gorham arrived, tends much to increase our confidence in statements which have thus had a double confirmation.

We could scarcely recapitulate M. Valleix's conclusions more briefly than he himself has done in the following summary:

"1st. In new-born children the pulse is less frequent than at the age of six months; the mean frequency while sleeping was 87; but sleep itself being a cause of diminished frequency of the pulse, it is necessary to select a somewhat higher figure as that of the normal pulse. Everything warrants the supposition that this varies in frequency from 90 to 100.

"2d. Increase of temperature, even when not so considerable as to be sensible to the subject of the observation, causes an appreciable acceleration of the pulse for each degree of temperature. Further observation alone can teach us what is the exact relation between increase of temperature and acceleration of the pulse.

"3d. Although the pulse constantly diminished in frequency in proportion as the day advanced, yet it would be premature to conclude that these facts support those which have been published by Dr. Guy. This author indeed states that the pulse progressively diminishes in frequency from morning to evening. Now, all my observations on children were made in the morning, and as the children were always fed before my observations commenced, the acceleration of the pulse in the early hours of the day, and its subsequent diminution in frequency must doubtless be in great measure attributed to this cause.

"4th. The least movement is sufficient to occasion a considerably increased frequency of the pulse in children, and the same result follows from any emotion or from mere fretfulness. Those authors who have examined the pulse without bearing in mind these circumstances have thus been unavoidably led to erroneous conclusions.

"5th. The influence of sex is very considerable even in young children. The pulse of little girls is in a marked degree more frequent than that of little boys.

"6th. The frequency of the pulse diminishes sensibly during sleep.

"7th. The frequency of the pulse does not undergo any very marked variations from the age of seven to twenty-seven months; its mean being about 126; on distinguishing the two sexes 121 in male children, 128 in female. These numbers express the frequency of the pulse under ordinary circumstances: but assuming the children to be in a state of perfect rest, the numbers would then be 119 in males, 124 in females.

"8th. Judging from some observations too few in number to be considered decisive, the pulse would appear to continue a little above 100 up to the age of six years.

"9th. The average frequency of inspiration in children between seven months and two and a half years old is 30 or 32; and is to the frequency of the pulse as 1·4." (p. 380.)

We regret much that M. Valleix did not test the accuracy of his statement, concerning the frequency of the pulse in new-born infants, by more extended observations. The average he obtained is considerably lower than that assumed as correct by Mr. Gorham; it is likewise lower than that of M. Billard, and than that mentioned by M. Roger as the result of his recent experiments. He grounds his opinion upon observations made at the Hospice des Enfans Trouvés on thirteen infants whose age varied from two days to twenty-one; a number decidedly too small to yield perfectly satisfactory results.

The fact, if fact it be, that the pulsations of the heart sink after birth permanently so far below their frequency during fetal life, and do not regain it till after the lapse of several months, is certainly very curious. Billard's observations tend to confirm the accuracy of this statement. He found that of 40 children, aged from 1 day to 10 there were as many in whom the pulse beat with about the same frequency as in the adult, as there were of those in whom it beat with much greater rapidity. The same fact, too, appeared to hold good in children aged from one to two months, and in others aged from two to three months, while in the small number of children above the age of one year, in whom Billard had ascertained the frequency of the pulse, he found that it beat with greater rapidity than in the adult. We cannot, however, feel thorough confidence in observations made at the Hôpital des Enfans Trouvés: for even the healthiest children there have been exposed to influences that must tend to impair their health before their admission to the institution, and others scarcely less injurious continue to act on them during their residence there. Eight of the thirteen subjects of M. Valleix's observations died a very few days afterwards; and though they appeared to him to be in perfect health when he investigated the frequency of their pulse, we must see his results confirmed before we can subscribe to their accuracy, or attempt to reason on them as on well-authenticated facts. The Lying-in Hospital at Dublin affords a wide field for examining the frequency of the pulse, during the first few days after birth, under circumstances the most likely to ensure trustworthy results; and we do hope that this point as well as that of the temperature of new-born infants, will engage the attention of the enlightened master of that institution.

The remainder of his statements rest on 567 observations, (made during 49 days, on 43 children, whose ages varied from 7 months to 6½ years) so accurately conducted as to warrant great confidence in their results. For the details of the observations we must refer to the paper itself, but we cannot avoid noticing the apparent disingenuousness of M. Valleix in not stating clearly that they are not new, and that this essay consists of little more than a more extended analysis of those researches on the pulse, which he published seven years ago in his 'Clinique des Maladies des Enfans.' He confesses to having had no opportunity of extending his researches on the pulse in new-born infants since the publication of that work, but no allusion is made to his observations on elder children having already appeared in print. On comparing them, however, with those which are contained in his former work, we find that the conclusions there were deduced from 461 observations made on 39 children, so that this essay consists in truth of the results of a further examination of the old materials, with the addition of 106 observations made on 5 subjects, who were examined at the same time with the others, though the results of his researches on them were not then published. We do not for a moment deny that this further examination of his former materials has led M. Valleix to many interesting and important conclusions. Still it would have been more ingenuous for M. Valleix to have stated the real nature of this paper, and to have announced that it contained those details for which he had no space in his work on Children's Diseases, but which he then promised "to publish elsewhere with all requisite minuteness."*

Lest we should be supposed to have misrepresented M. Valleix, we quote the words in which in the preface to this volume he speaks of the essay we have been noticing.

> "In the second memoir," says he, "I have related my observations on the pulse in children. Considering this question as very complex, I was anxious to see whether by subdividing it and looking at it in all points of view, it would be possible to arrive at more trust-worthy conclusions, than are attained to by the ordinary modes of investigation. This undertaking has impressed on me the conviction that the way I have pursued is the only one which can lead to truth." (p. 25.)

But pass we now to a memoir by M. Ducrest which stands next in order. "On an accidental ossification discovered on the internal surface of the cranium in puerperal women." M. Valleix informs us that M. Ducrest, studied this singular alteration, of the occurrence of which some

* Clinique des Maladies des Enfans Nouveau-Nés, p. 23.

German writers had a kind of vague notion, "*à peine entrevu par quelques médecins Allemands,*" in the different periods of its formation. M. Ducrest, himself remarks.

"It would have been of great service to me to have met with any work on the same subject that might have enabled me to add to the rather low figures on which some of my conclusions rest. I regret therefore that I was unable to procure a memoir, the publication of which in the German journals had been mentioned to me by M. Danyau." (p. 381.)

Who would have thought, from this language that a minute account of the appearance discribed by M. Ducrest as the results of 90 observations has been already published by M. Rokitansky: first in the 'Oesterr. Med. Jahrb.,' Bd. xv, Stück iv; and subsequently in his 'Pathologische Anatomie,' Bd. ii, Liefer. ii, s. 237?

In some respects indeed this very ignorance of the researches of others, confers additional value on the observations of M. Ducrest, which have led him wholly unawares to conclusions very similar in all the more important respects to those of Rokitansky. He sums up his paper in the following brief statement.

"1st. That on the internal surface of the cranium of lying-in women, we meet with an accidental product, at first analogous to cartilage, and subsequently assuming the consistence of bone.

"2d. That the cranium and dura mater in connexion with it, exhibit no alteration.

"3d. That it develops itself most frequently in young women.

"4th. That its presence does not give rise to any peculiar symptoms." (p. 432.)

These conclusions agree pretty nearly with those of M. Rokitansky; there are, however, some points of difference in his description of the affection which we will presently mention. After detailing in full twelve observations M. Ducrest proceeds to give a general description of this pathological condition. The first trace of it consists in a somewhat increased vascularity of certain points on the interior of the skull; usually of some two or three of the digital fossæ; while the bone and dura mater continue perfectly unchanged. After a time a red spot is seen in the midst of each of these little patches of vascular network; and on examination it is found to consist of red pulpy matter easily removed by the finger. When the number of these spots was very small, not exceeding two or three, they were confined to one side of the cranium, but if more numerous they existed on both sides. In 43 out of 90 cases they existed only on the vault of the cranium, and were confined to the bottom of the digital fossæ; 38 times they were also present along the lateral or anterior parts of the frontal bone; twice they existed on the parietal bones, and thrice along the sagittal suture. When more numerous they cover the eminences between the fossæ, and, uniting together, form a continuous layer of variable extent. This union usually commences by the fusion of the patches on either side of the frontal bone, whence the deposit of new matter extends in various directions. When the deposit upon the vault of the cranium is very extensive, the base itself becomes involved; the anterior or lateral part of the roof of the orbits, or the upper surface of the processes of Ingrassias being first affected, and that portion of the occiput on which the cerebellum rests the last. In a still more advanced stage, the whole arch is covered, but the new deposit never extends completely over the base. A similar deposit on the exterior of the skull existed only in four instances, and did not then correspond in situation to the osteophyte within.

These deposits vary in thickness from that of a couple of sheets of paper to three or four millimeters. Their thickness is by no means in direct proportion to their extent, nor is it the same through the whole extent of the layer; but is usually thickest anteriorly, less thick behind in all those instances where the deposit involves the whole arch of the cranium. When the patches of new matter have advanced beyond their first stage of development, they are seen to be formed of two layers: an internal, meningeal, which is thin, compact, and represents the superficial lamina of bones; and an external, cranial, which is thicker and cellular, representing their spongy substance. The dura mater, in contact with these growths, always appears perfectly natural, the vascularity of the cranium around them is considerably increased, but nothing seems to warrant the supposition that they are the result of an inflammatory process.

No symptoms peculiar to this affection existed during life, and though it appears to be connected with pregnancy, yet the circumstance of its having come under M. Ducrest's observation more frequently in primiparæ than in women who had previously borne children, renders the exact nature of this connexion exceedingly difficult to discover.

Professor Rokitansky's statements complete this account, and confirm it in most points. According to him, however, the new deposits are found on the prominences of the inner surface of the cranium more frequently than in the depressions. His account of their formation agrees with that of M. Ducrest, and like him he distinguishes three different stages in their development. In its first stage the new matter exists as a mere vascular exudation, easily removed from the inner table of the skull, which is either perfectly normal in appearance, or, at most, has lost a little of its smoothness. In its second stage, it is a soft, flexible, finely porous cartilaginous lamina, beneath which the inner table of the skull presents a manifest roughness, or at least the pores of its vessels are seen to be notably enlarged. At the commencement of the third stage it is still pliable, but is no longer wholly cartilaginous: bony matter being now intermingled in its substance. It presents two surfaces: one smooth and finely porous, which is in contact with the dura mater; the other, which is firmly connected with the inner table of the skull, is rough and cellular. Numerous lamellæ and spiculæ of this spongy substance are the medium of its connexion with the cranium, and both they and many blood-vessels are torn through in any attempt to detach it.

The new deposit does not pass beyond this stage during pregnancy or the puerperal state, but Professor Rokitansky has followed it further than M. Ducrest, and is able to state positively that it subsequently becomes completely ossified, and forms an integral portion of the skull, either becoming exceedingly dense and one in substance with the inner table of the skull, or else being connected with it through the medium of a new diploe. In successive pregnancies this deposit becomes thickened by the addition of new layers, which, however, are not immediately connected with the old bone, but a thin stratum of diploe always intervenes between the two.

With reference to the causes of the affection, Professor Rokitansky is unable to go beyond the mere statement, that a puerperal hyperostosis of the skull exists in connexion with repeated pregnancies; his experience, contrary to that of M. Ducrest, having led him to regard it as of more frequent occurrence in multiparæ than in primiparæ. He has met with it in women dying at all periods of pregnancy, from the third month to its termination, as well as in those who died in the puerperal state. It has not appeared to him to betray its existence by any peculiar symptom or to predispose to any particular form of fatal illness.

Professor Rokitansky mentions also that he has met with a similar condition in eighteen other persons, some of whom were males, though in these cases the deposit of bone was by no means so extensive as in pregnant or puerperal women. These persons, too, had died of most various diseases, and presented no symptom during life indicative of the existence of this affection.

The last paper is by M. Flauvel, on 'Capillary Bronchitis;' and consists of a republication of his dissertation which treated of this affection in children, and of a series of observations upon it as it occurs in the adult. In our Number for October 1841, will be found a review of his dissertation, which renders any notice of one half of his essay needless; and we reserve for some future time our notice of his observations on the disease in adults, since it would engage us in discussion for which we have no space at present. In brief, then, be it said, that they show the same conscientious pains-taking observation as we noticed with well-merited praise in his inaugural dissertation.

Such, then, are the contents of the second volume of the Transactions of a society which proposed to itself to reform, almost to remodel, medical science, and which has interposed a delay of six years between the publication of the first and second volume of its memoirs, in order to ensure their excellence. The three essays we have noticed contain, unquestionably, much that is very valuable; but the pretensions of the society do not allow us to judge their performances by any ordinary standard. We find that the whole of one essay, and half of another, consist of little more than a republication, in a more extended form, of facts which their authors have long since laid before the profession; while the third contains an account of a phenomenon described more completely several years ago by a German anatomist.

We honestly confess that we have risen from the perusal of this volume with disappointment. This is not the place for us to examine how far the Société d'Observation are in the right course in their mode of interrogating nature; sure we are, however, that their memoirs must differ from those we have just noticed, before we can look for results at all commensurate with their expectations.

